# Catalpol Protects Against Spinal Cord Injury in Mice Through Regulating MicroRNA-142-Mediated HMGB1/TLR4/NF-κB Signaling Pathway

**DOI:** 10.3389/fphar.2020.630222

**Published:** 2021-02-08

**Authors:** Hougang Xia, Dandan Wang, Xiaohui Guo, Kaidi Wu, Fuwei Huang, Yanjiang Feng

**Affiliations:** ^1^ Department of Rehabilitation Medicine, Luoyang Orthopedic-Traumatological Hospital of Henan Province (Henan Provincial Orthopedic Hospital), Luoyang, China; ^2^ Department of Nursing, Luoyang Orthopedic-Traumatological Hospital of Henan Province (Henan Provincial Orthopedic Hospital), Luoyang, China; ^3^ Department of Spinal Surgery, Luoyang Orthopedic-Traumatological Hospital of Henan Province (Henan Provincial Orthopedic Hospital), Luoyang, China

**Keywords:** spinal cord injury, catalpol, inflammation, apoptosis, microRNA-142, HMGB1/TLR4/NF-κB signaling pathway

## Abstract

**Background:** Spinal cord injury (SCI) is a devastating condition that leads to paralysis, disability and even death in severe cases. Inflammation, apoptosis and oxidative stress in neurons are key pathogenic processes in SCI. Catalpol (CTP), an iridoid glycoside extracted from *Rehmannia glutinosa*, has many pharmacological activities, such as anti-inflammatory, anti-oxidative and anti-apoptotic properties.

**Purpose:** Here, we investigated whether CTP could exert neuroprotective effects against SCI, and explored the underlying mechanism involved.

**Methods:** SCI was induced by a weight-drop device and treated with CTP (10 mg and 60 mg/kg). Then the locomotor function of SCI mice was evaluated by the BBB scores, spinal cord edema was measured by the wet/dry weight method, oxidative stress markers and inflammatory factors were detected by commercial kits and neuronal death was measured by TUNEL staining. Moreover, the microRNA expression profile in spinal cords from mice following SCI was analyzed using miRNA microarray. In addition, reactive oxygen species (ROS) generation, inflammatory response and cell apoptosis were detected in murine microglia BV2 cells under oxygen-glucose deprivation (OGD) and CTPtreatment.

**Results:** Our data showed that CTP treatment could improve the functional recovery, as well as suppress the apoptosis, alleviate inflammatory and oxidative response in SCI mice. In addition, CTP was found to be up-regulated miR-142 and the protective effects of CTP on apoptosis, inflammatory and oxidative response may relate to its regulation of HMGB1/TLR4/NF-κB pathway through miR-142.

**Conclusion:** Our findings suggest that CTP may protect the spinal cord from SCI by suppression of apoptosis, oxidative stress and inflammatory response via miR-142/HMGB1/TLR4/NF-κB pathway.

## Introduction

Spinal cord injury (SCI) is one of the most devastating traumatic lesions, which can result in permanent disability, or loss of movement and sensation in patients (Lam et al., 2015). In the USA, approximately 12,000 persons suffer traumatic SCI each year and brings $40.5 billion per year in costs (DeVivo et al., 2002). The pathogenesis of SCI comprises two stages: primary injury and secondary injury, the former is mainly caused by direct injury to the spinal cord and it is irreversible, whereas the latter is induced by excessive inflammatory response, apoptosis and oxidative stress and it is preventable or reversible (Blight, 1985; Goldshmit et al., 2015; Orr and Gensel, 2018). Therefore, novel and effective therapeutic strategies to improve pathophysiological changes of secondary injury have gained extensive attention.

Catalpol (CTP) is a natural iridoid glycoside compound that is found in *Rehmannia glutinosa*. Several studies demonstrated that CTP has anti-apoptotic, anti-inflammatory and antioxidant effects, as well as neuro-protective effects in various types of diseases (Zhang et al., 2007; Hu et al., 2010; Bi et al., 2013; Choi et al., 2013). For example, CTP exerts a neuroprotective effect in the MPTP mouse model of Parkinson’s disease through reducing the pro-inflammatory factors and inflammasome (Wang et al., 2019a). Wang et al. showed that CTP ameliorated the degeneration of cholinergic neurons via elevating brain-derived neurotrophic factors (Wang et al., 2009). Wang et al. found that CTP could alleviate chronic constriction injury through suppressing neuroinflammation in a rat model (Wang et al., 2014). Zhu et al. reported that CTP improved axonal outgrowth and reinnervation of injured sciatic nerve in mice by activating Akt/mTOR pathway (Zhu et al., 2019b). Liu et al. demonstrated that CTP pretreatment alleviated cerebral ischemia/reperfusion (CI/R) injury through enhancing atpase activity in gerbil (Liu et al., 2014). However, whether CTP has a protective effect on SCI remains unclear.

MicroRNAs (miRNAs) are single-stranded non-coding RNAs that negatively regulate gene expression by binding to the 3ʹ-UTR of their target genes at the post-transcriptional level (Ambros, 2004). To date, several miRNAs have been detected in the spinal cord, and are involved in the pathophysiology of SCI, such as inflammation, oxidation and apoptosis. For example, Feng et al. demonstrated that miR-204 was downregulated in the serum from SCI patients, and miR-204 overexpression promoted SCI recovery through inhibiting inflammatory response in SCI mice (Feng et al., 2019). Boris Sabirzhanov et al. showed that inhibition of miR-711 limited tissue damage and motor dysfunction after contusive SCI in mice (Sabirzhanov et al., 2019). Wan et al. revealed that miR-129-5p alleviated spinal cord injury in mice via suppressing the apoptosis and inflammatory response through HMGB1/TLR4/NF-kappaB pathway (Wan et al., 2020). Several studies have confirmed that the effect of CTP in the prevention or treatment of various diseases, is mediated by miRNAs and their target genes (Zhu et al., 2019a; Zhang et al., 2020). For example, Zou et al. demonstrated that CTP improved myocardial damage by regulating miR-140-5p/HDAC4 axis in diabetic cardiomyopathy (DCM) mice (Zou et al., 2019). Zhu et al. found that CTP could affect miR-124 to regulate PI3K/AKT/mTOR pathway, thus promoting axonal growth in stroke rat (Wu et al., 2013). Against this background, we were interested in determining whether miRNAs are involved in the therapeutic effect of CTP on SCI.

In the current study, we used a mouse model of SCI to study the therapeutic effects of CTP and addressed the molecular mechanism by identifying CTP-induced miRNA dysregulations. Our results suggested that CTP exerts the protective effects on SCI via mediating miR-142/HGMB1/TLR4/NF-κB signaling pathway and act as a potential therapeutic agent for the treatment of SCI.

## Materials and Methods

### Animals and Treatments

Adult female C57BL/6 mice (10–12 weeks, 20–25 g) were purchased from Shanghai SLAC Laboratory Animal Co., Ltd. (China). Mice were housed under standard conditions (12:12 light-dark cycle light–dark cycle, 24 ± 2°C, ∼40% humidity) with free access to food and water. All animal care and experimental procedures were performed at the Animal Experimental Center of Luoyang Orthopedic-Traumatological Hospital of Henan Province and all procedures were approved by the Ethics Committee for Experimental Animals of Luoyang Orthopedic-Traumatological Hospital of Henan Province (Ethical approval No.: 2018-000115).

### Experimental Design

Mice were randomly divided into four groups (n = 6/group): 1) the Sham group; 2) SCI group; 3) SCI + CTP group (10 mg/kg); 4) SCI + CTP group (60 mg/kg). Mice were anesthetized with an intraperitoneal (i.p.) injection of 50 mg/kg pentobarbital sodium (Sigma-Aldrich, St. Louis, MO, U.S.A.). SCI model was established using the Infinite Horizon spinal cord impactor (Precision Systems and Instrumentation) as previously described (Wu et al., 2013). On the following day after SCI, CTP (10 or 60 mg/kg, Sigma-Aldrich, St. Louis, MO, U.S.A.) was treated to mice once a day for 4 weeks by gavage. Mice that underwent all surgical procedures without inflicted crush injury were used as Sham group.

In another test, mice were randomly divided into four groups: SCI group, SCI + CTP group, SCI + CTP + antagomiR-142 group, and SCI + CTP + antagomiR-negative control (NC) group. In the SCI + CTP + antagomiR-142 group/antagomir-NC group (n = 6/group), the mice were subjected to SCI and then treated with antagomiR-142/antagomir-NC (5 nmol/g/day, RiboBio, Guangzhou, China) via intrathecal injection starting 15 min after SCI for three consecutive days (on day 1 to day 3). The injection rate was 0.2 μL/min. On the following day after SCI, CTP (60 mg/kg) was treated to mice once a day for 4 weeks by gavage.

At 28 days post-injury, mice were humanely killed with i.p. injection of 50 mg/kg pentobarbital sodium followed by cervical dislocation, and subsequently, the spinal cord tissues (a 10 mm segment containing the injury epicenter) was harvested, and then fixed in 4% paraformaldehyde (PFA) (4° C) in PBS (pH 7.4) for 20 min, embedded in paraffin, and sectioned at 4 μm thickness for subsequent experiments.

### Basso, Beattie and Bresnahan Score

The locomotor activity was evaluated using the BBB score method at 1, 7, 14, 21, and 28 days after SCI as described previously (Basso et al., 1995). The scores were recorded by two independent and well-trained investigators according to the BBB scales.

### Spinal Cord Water Content Measurement

At 28 days post-injury, the spinal tissues obtained from experimental mice were immediately weighted, and then dried at 80° C for 48 h. After the dry weight was measured, the spinal cord water content was evaluated as follows: water content = [(wet weight - dry weight)/wet weight] ×100%.

### TUNEL Assay

At 28 days post-injury, the spinal tissues obtained from experimental mice were fixed in 4% paraformaldehyde (PFA) (4° C) in PBS (pH 7.4) for 20 min, embedded in paraffin, and sectioned at 4 μm thickness. Then, the apoptosis in the spinal tissues was detected by One Step TUNEL Apoptosis Assay Kit (Beyotime Biotech., Jiangsu, China), TUNEL positive cells were observed under fluorescence microscopy (HB050; Zeiss, Hamburg, Germany) (×200 magnification).

### Detection of Reactive Oxygen Species

ROS generation was calculated using 2,7-dichlorodihydrofluorescein diacetate (2,7-DCF-DA) staining kit (cat no. S0033S, Beyotime Biotech., Jiangsu, China) according to the manufacturer’s instructions. Fluorescence images were captured and analyzed using an inverted fluorescence microscope (×200 magnification).

### MDA and SOD Activity Assay

MDA (cat. no. S0131S) and SOD (cat. no. S0086) activities in tissue grinding fluid from mice and cell supernatants were measured by commercial kits (Beyotime Biotech., Jiangsu, China) according to the manufacturer’s instructions.

### ELISA Assay

The protein expressions of TNF-α (cat. no. PT516), IL-6 (cat. no. P1328), IL-1β (cat. no. P1303) and IL-10 (cat. no. PI525) in tissue grinding fluid from mice and cell supernatants were measured by ELISA, using protocols supplied by the manufacturer (Beyotime Biotech., Jiangsu, China).

### Microarray Assay

Microarray dataset was obtained from GEO database (https://www.ncbi.nlm.nih.gov/geo/query/acc.cgi?acc=GSE19890) and the GEO accession number is GSE19890. GEO2R (www.ncbi.nlm.nih.gov/geo/geo2r/), an interactive web tool was applied to compare the samples in two different groups under the same experimental condition. Differentially expressed miRNAs (DE-miRNAs) were then identified based on the fold change. The heat map of DE-miRNAs was created using a method of hierarchical clustering by GeneSpring GX, version 7.3 (Agilent Technologies, California, United Stages).

### Real-Time Quantitative PCR

Total RNA from spinal cord tissues and cells was isolated using a mirVana™ miRNA Isolation Kit (Thermo Fisher Scientific) as the manufacturer’s instructions. Reverse transcription of miRNA and mRNA was generated by using PrimeScript RT Reagent Kit (Takara Biotech, Dalian) and an iScript cDNA synthesis kit (Bio-Rad), respectively. qRT-PCR was performed using the SYBR Premix Ex Taq (TaKaRa, Tokyo, Japan) on an ABI Prism7500 Sequence Detection System (Thermo Fisher Scientific). U6 was used as an internal control for miRNAs, and GAPDH for HGMB1. The primers used were as follows: HMGB1 forward: 5′-CCA​ACA​GGC​AAA​TGG​GGT​CT-3′; reverse: 5′-TAA​CTG​GTG​GGC​CAG​GGA​TA-3′; GAPDH forward: 5′-TCA​ACG​ACC​CCT​TCA​TTG​ACC-3′, reverse: 5′-CTT​CCC​GTT​GAT​GAC​AAG​CTT​C-3′; miR-223 forward: 5′-TGG​CTG​TCA​GTT​TGT​CAA​AT-3′, reverse: 5′-GTG​CAG​GGT​CCG​AGG​T-3′; miR-126 forward: 5′-GTC​GTA​TCC​AGT​GCA​GGG​TCC​GAG-3′, reverse: 5′-GTC​GTA​TCC​AGT​GCA​GGG​TCC​GAG-3′; miR-124 forward: 5′-TCG​TTA​AGG​CAC​GCG​GTG-3′, reverse: 5′-GTG​CAG​GGT​CCG​AGG​T-3′; miR-21 forward: 5′-GCA​GGG​TCC​GAG​GTA​TTC-3′, reverse: 5′-CTA​CTC​ACA​AAA​CAG​GAG​TGG​AAT​C-3′; miR-494 forward: 5′-TGG​TGA​TGG​GAT​TTG​AAA​CAT​ACA​CGG​GAA​AC-3′, reverse: 5′-AGA​TAG​ACG​G-TGT​CGC​TGT​TGA​AGT​CAG-3′; miR-210 forward: 5′-ACA​CTC​CAG​CTG​GGA​GCC​CCT​GCC​CAC​CGC-3′, reverse: 5′-TGG​TGT​CGT​GGA​GTC​G-3′; miR-142 forward, 5′-AAC​TCC​AGC​TGG​TCC​TTA​G-3′ and reverse, 5′-TCT​TGA​ACC​CTC​ATC​CTG​T-3′. U6, forward: 5′-CTC​GCT​TCG​GCA​GCA​CA-3′, reverse: 5′-GTC​ATA​CTC​CTG​CTT​GCT​GAT-3′. Analyses of gene expression were performed by the 2^−ΔΔ^Ct method (Livak and Schmittgen, 2001).

### Cell Culture and Treatment

Immortal BV-2 murine microglial cell was obtained from ATCC (Manassas, VA, U.S.A.) and cultured in DMEM/F12 supplemented with 10% FBS (Gibco BRL, Grand Island, NY), and 1% penicillin and streptomycin (Sigma, St. Louis, MO) in 5% CO_2_ at 37° C.

An SCI model in BV-2 cells was established according to previous study. and BV-2 cells without any treatment were used as the control group. The prepared BV-2 cells were cultured in glucose-free medium under hypoxic condition (5% CO_2_, 0.2% O_2_) for 6 h (oxygen and glucose deprivation [OGD]) and then, cells were subjected to re-oxygenization for 12 h to construct OGD/reoxygenation (OGD/R) injury cell model.

### Cell Transfection

When BV-2 cells were grown to 70% confluent, transfections of 20 nM antagomiR-142/antagomir-NC were performed by using Lipofectamine 2000 (Invitrogen, Thermo Fisher Scientific, Inc.) according to the manufacturer’s instructions.

### Cell Viability Analysis

Cell viability was measured using the Cell Counting Kit-8 (CCK-8, Beyotime Institute of Biotechnology, Shanghai, China) assay according to the manufacturer’s instruction. The BV-2 cells (5 × 10^4^ cells/well) were seeded in 96-well plate. After 24 h incubation, CTP (0, 5, 10 µM) were added into cells, and then cells were further subjected to OGD/R injury. After 24 h, 10 μL CCK-8 reagent was added to each well and continuously cultured for another 4 h. The absorbance rate at 450 nm was measured by Microplate Reader (Bio-Rad, U.S.A.).

### Flow Cytometry Analysis and Caspase-3 Assay

The BV-2 cells were fixed in 70% ice-cold methanol at 4°C for 30 min, and then stained with 5 μL of AnnexinV-FITC and 1 μL of propidium iodide (PI, 50 μg/ml in PBS). After 15 min incubation at room temperature in darkness, the apoptosis was measured on FACScan flow cytometer (Beckman Coulter, Inc., Brea, CA, USA) and then analyzed by FlowJo 8.7.1 software (Ashland, OR).

Caspase-3 activity was assessed by a caspase-3 activity assay kit (Beyotime Institute of Biotechnology, Shanghai, China) according to the manufacturer’s instructions.

### Luciferase Reporter Assay

The 3′-UTR of HMGB1 and the mutated sequence were inserted into the pGL3 control vector (Promega Corporation) to construct the wild-type (wt) HMGB1-3′-UTR vector and mutant HMGB1-3′-UTR vector, respectively. BV-2 cells were transfected with antagomiR-142/antagomir-NC and these luciferase reporter plasmids using Lipofectamine 2000 (Invitrogen). At 48 h post-transfection, luciferase activity was assessed using the dual luciferase reporter kit (Promega Corporation). Renilla activity was used to normalize firefly luciferase activity.

### Western Blot

Total protein was obtained using RIPA lysis buffer (Beyotime Biotechnology, Shanghai, China) and quantified with a BCA protein assay kit (Pierce; Thermo Fisher Scientific, Inc.). Next, the proteins in the lysates were separated by SDS-PAGE gels and transferred to PVDF membranes (GE Healthcare) followed by incubation in a 5% skim milk solution for 1 h at room temperature. Subsequently, the specific primary antibodies were incubated in the membranes at 4°C overnight, including HMGB1 (#6893, 1:1,000 dilution), TLR4 (#14358, 1:1,000 dilution), Myd88 (#4238, 1:1,000 dilution) IκB-α (#4812, 1:1,000 dilution), phospho-IκB-α (#2859, 1:1,000 dilution), phospho-NF-κB p65 (#3033, 1:1,000 dilution), total p65 (#8245, 1:1,000 dilution) and β-actin (#3700, 1:1,000 dilution) (Cell Signaling Technology). Subsequently, the corresponding anti-rabbit secondary antibodies (cat no.3678, 1:2,000) were added into the membranes for 2 h at room temperature. The protein band was detected by chemiluminescence with Pierce ECL kits (Millipore). Semi-quantification was performed using ImageJ version 1.46 (MD, USA). The original images of Western Blot are uploaded as Supplementary Material.

### Statistical Analysis

Statistical analysis was conducted using GraphPad Prism (version 5.0, Inc., La Jolla, CA, U.S.A.). Data are expressed as mean ± SD. Differences between groups were determined by one-way analysis of variance followed by least significant difference post-hoc tests. Unpaired Student’s t test was used for the comparison of two groups. Differences were considered statistically significant when *p* < 0.05.

## Results

### Catalpol Improves Recovery of Spinal Cord Injury Mice by Reducing Apoptosis

**FIGURE 1:**
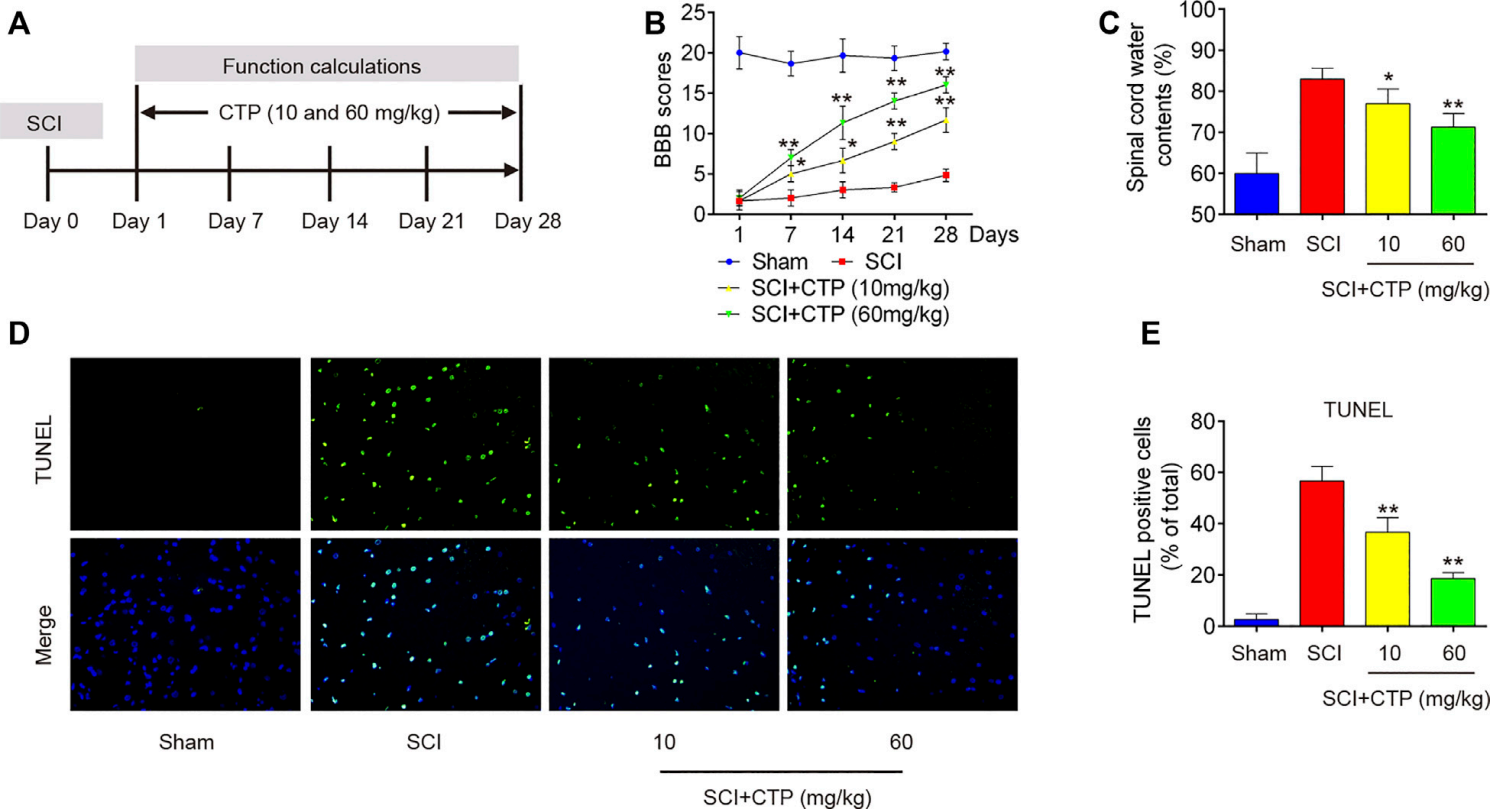
Catalpol improves recovery of SCI operated mice by reducing apoptosis. After SCI mice model was established, CTP (10 and 60 mg/kg) was subjected to SCI mice once a day for 28 days. **(A)** SCI mice model and Catalpol treatment protocol. **(B)** Changes in BBB scores of SCI mice treated with or without Catalpol at the indicated days. **(C)** Water content in spinal cord of mice was detected. **(D–E)** TUNEL staining of neuronal apoptosis of spinal cord tissues post-Catalpol treatment at 28 days. Data represent the mean ± SD of three independent experiments. *p < 0.05, **p < 0.01 vs. SCI group.

First, we established a SCI mice model as above described, and then CTP (10 and 60 mg/kg) was subjected to SCI mice by gavage once a day for 28 days (Figure 1A). We found that BBB scores were significantly lower in the SCI group (*p* < 0.0001) than that in Sham group (Figure 1B). The water contents in spinal cord of SCI mice (*p* = 0.0021) were significantly increased compared with Sham group (Figure 1C). Moreover, the number of TUNEL positive cells was notably increased in spinal cord tissues of SCI mice (*p* = 0.0001) (Figures 1D,E). All these findings suggest the successful construction of mouse SCI model. After CTP (10 and 60 mg/kg) treatment, our results showed the obvious increase of BBB scores, notable reduction of water contents (*p* = 0.0408; 0.0083) and TUNEL positive cell number (*p* = 0.0093; 0.0005) compared with SCI group (Figures 1B–E). It was also observed that the potency of 60 mg/kg CTP was better than the group of 10 mg/kg CTP, which were close to control. In a word, these results suggested that CTP could relieve SCI by reducing neuronal apoptosis in mice.

### Catalpol Reduces Inflammation and Oxidative Stress in Spinal Cord of Spinal Cord Injury Mice

**FIGURE 2:**
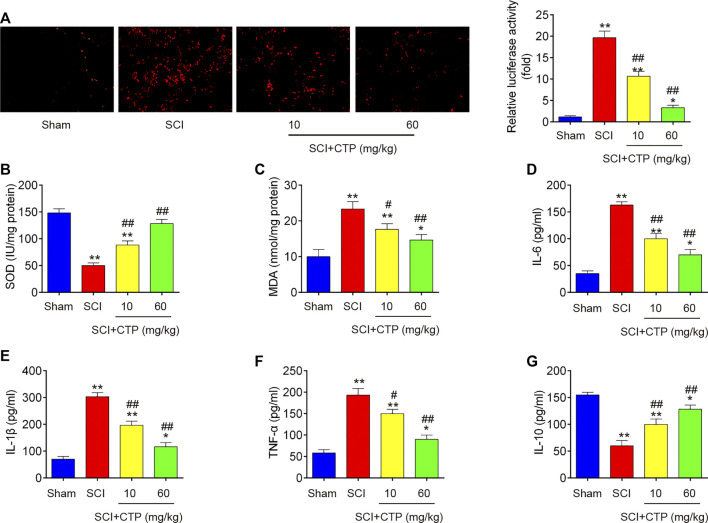
Catalpol reduces oxidative stress and inflammation in spinal cord of SCI mice. After SCI mice model was established, CTP (10 and 60 mg/kg) was subjected to SCI mice once a day for 28 days. **(A)** ROS generation was calculated using 2,7-DCF-DA staining kit. **(B and C)** The contents of MDA and SOD were measured by commercial kits. **(D–G)** The inflammatory cytokines including TNF-α, IL-1β, IL-6 and IL-10, were evaluated by ELISA assays. Data represent the mean ± SD of three independent experiments. *p < 0.05, **p < 0.01 vs. Sham group; #*p* < 0.05, ##*p* < 0.01 vs. SCI group.

During secondary injury, excessive oxidative stress and inflammation in neuron are two major pathogenic processes. Given the anti-apoptotic, anti-inflammatory and antioxidant effects of CTP, we explored whether CTP could improve the secondary injury via regulating these pathogenic processes. First, the alterations in oxidative stress were investigated. As shown in Figure 2A, the ROS levels in SCI group (*p* < 0.0001) was significantly increased compared with Sham group. In contrast, the ROS levels in CTP groups (*p* = 0.0092; *p* < 0.0001) was lower than that of SCI group. Meanwhile, the levels of SOD in SCI mice (*p* < 0.0001) were markedly decreased, and MDA levels were significantly increased (*p* = 0.0013) compared with Sham group. However, the levels of SOD (*p* = 0.0019; *p* = 0.0001) were increased and the levels of MDA (*p* = 0.0191; *p* = 0.0044) was decreased in spinal cord tissues of CTP-treated mice compared with SCI group (Figures 2B,C). Moreover, the releases of TNF-α, IL-6, IL-1β, and IL-10 in spinal cord tissues of SCI mice were also detected by ELISA. Compared with the Sham group, the levels of TNF-α (*p* = 0.0002), IL-6 (*p* < 0.0001) and IL-1β (*p* < 0.0001) were increased, and the important anti-inflammatory cytokine, IL-10 (*p* = 0.0001) was markedly decreased. However, Catalpol treatment significantly reduced the levels of these pro-inflammatory cytokines (TNF-α, *p* = 0.0147 or *p* = 0.0006; IL-6, *p* = 0.0007 or *p* = 0.0002; IL-1β, *p* = 0.0010 or *p* = 0.0001), while IL-10 levels (*p* = 0.0080 or *p* = 0.0007) were enhanced (Figures 2D–G). Collectively, these data indicate that Catalpol could relieve SCI in mice by reducing inflammation and oxidative stress.

### MiR-142 Was Upregulated by Catalpol Treatment in Spinal Cord Tissue of Spinal Cord Injury Mice

**FIGURE 3:**
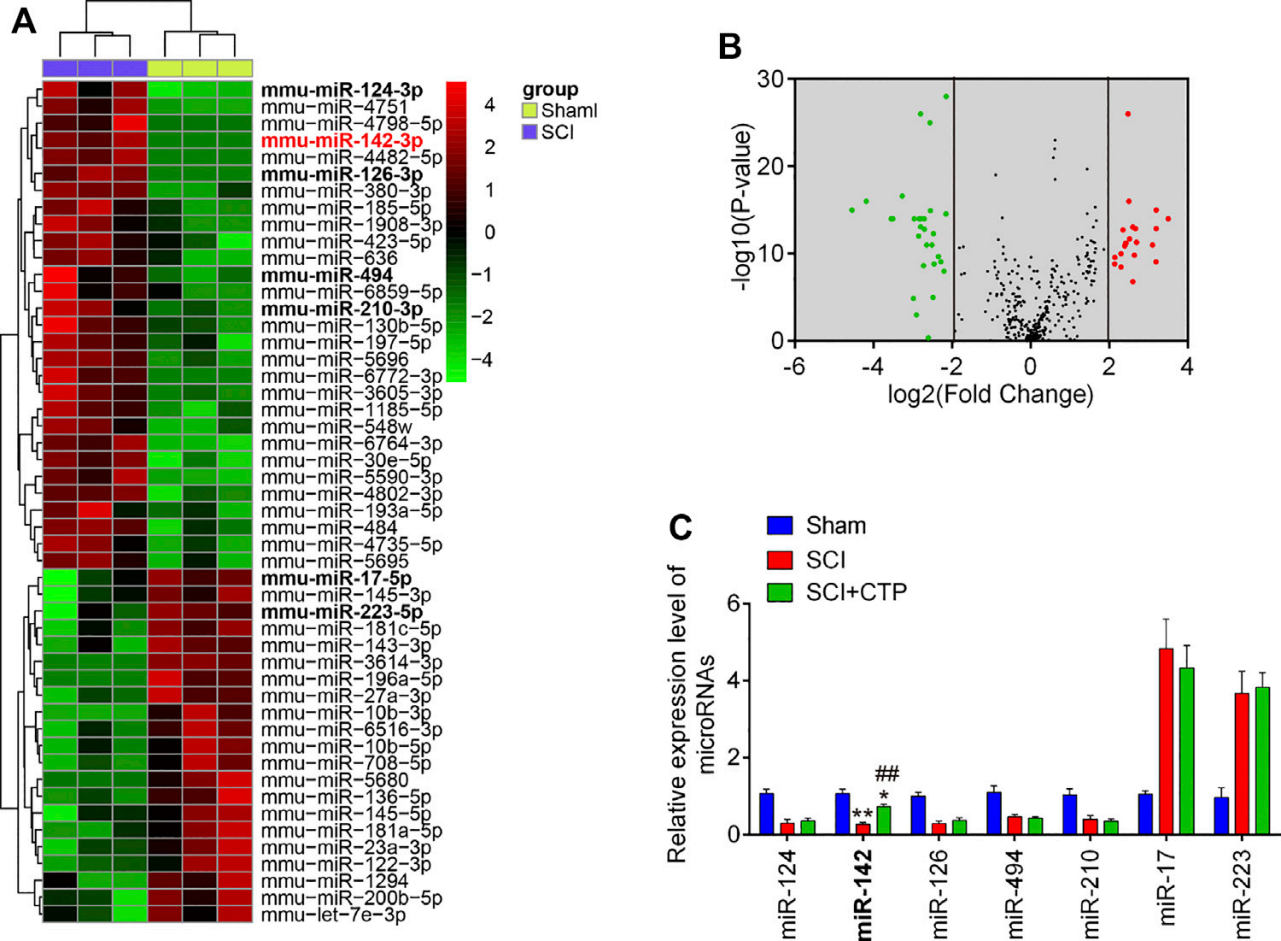
Catalpol upregulated the miR-142 expression in SCI mice. **(A)** Heatmap of normalized expression levels of miRNAs in SCI mice and Sham mice (n = 3/group). Microarray dataset was obtained from GEO database and the GEO accession number is GSE19890. Green indicates low expression levels, while red indicates high expression levels. **(B)** Volcano plot presenting the differentially expressed miRs. *Y*-axis represents log transformed *p*-value, and *x*-axis indicates the mean expression differences of miRs between SCI mice and Sham mice. |log2FoldChange| >2 was set as the cut-off criteria. **(C)** The expression levels of miR-124, miR-142, miR-126, miR-494, miR-210, miR-17 and miR-223 were detected in Sham, SCI, and SCI + CTP groups by qRT-PCR. Data represent the mean ± SD of three independent experiments. *p < 0.05, **p < 0.01 vs. Sham group; ##*p* < 0.01 vs. SCI group.

Given the key roles of miRNAs during secondary injury of SCI, we investigate whether CTP exerts the protective effects through regulation of miRNAs. Via retrieving the microarray dataset obtained from GSE19890, total of 50 differentially expressed miRNAs (29 miRNAs were significantly downregulated and 21 miRNAs were upregulated) in spinal cord tissues were screened out (Figures 3A,B). To confirm which miRNA could be affected by CTP, we selected seven miRNAs(miR-124, miR-126, miR-494, miR-210, miR-142, miR-17 and miR-223) that have been widely studies in SCI (Ujigo et al., 2014; Hu et al., 2015; Liu et al., 2015; Zhu et al., 2017; Song et al., 2019; Zhang et al., 2019). The results showed that miR-17 and miR-223 were markedly decreased, while miR-124, miR-126, miR-494, miR-210 and miR-142 were significantly increased, which are in line with previous reports, suggesting the experimental reliability of our microarray results. As shown in Figure 3C, only miR-142 was significantly increased by CTP (*p* = 0.0006) which was selected as the target miRNA for further studies.

### Catalpol Reduces the Apoptosis, Inflammation and Oxidative Stress in Spinal Cord Injury Mice Through Promoting MiR-142

**FIGURE 4:**
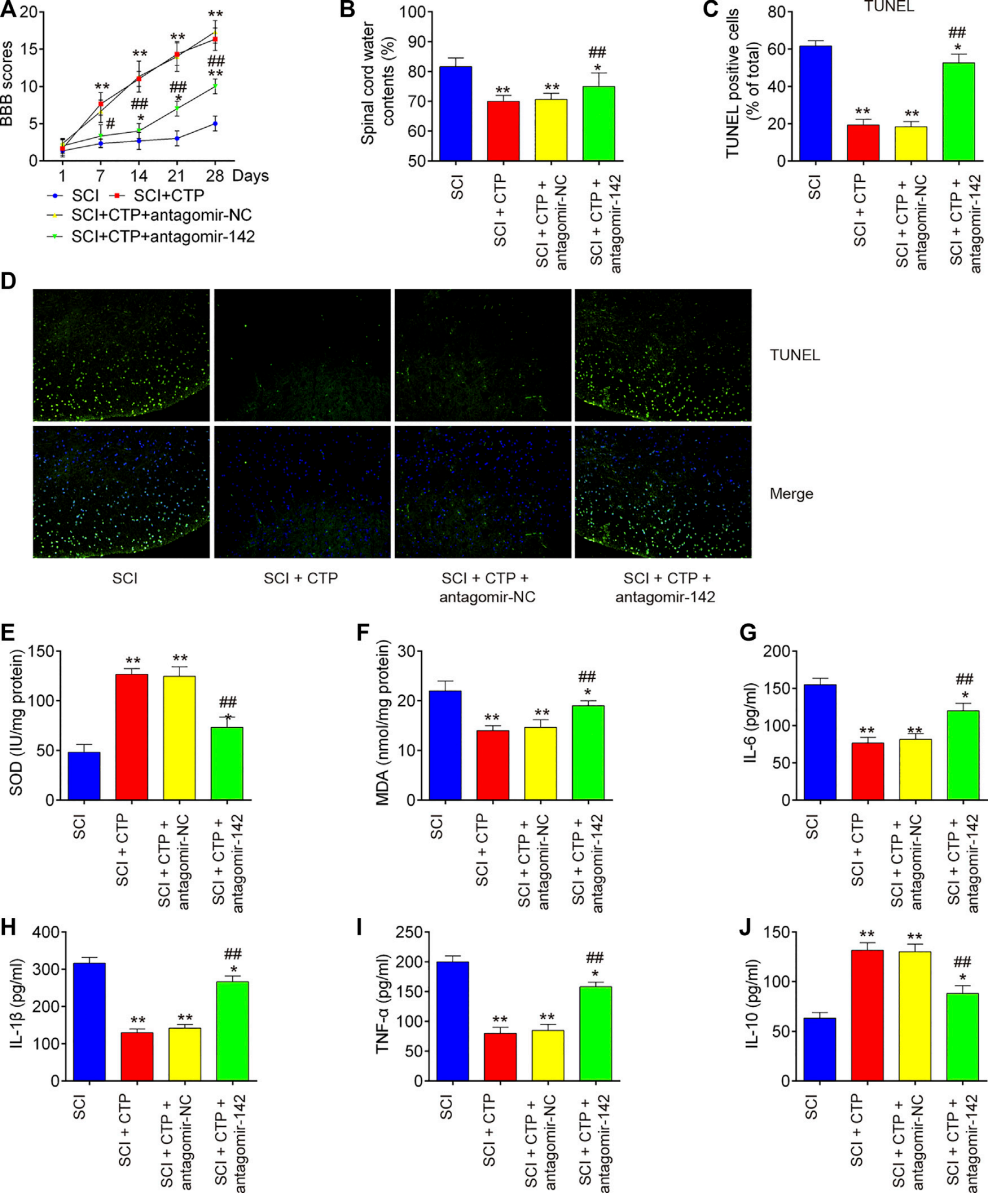
Catalpol reduces the apoptosis, oxidative stress and inflammation in SCI mice through promoting miR-142. The mice were subjected to SCI and then treated with antagomiR-142/antagomir-NC via intrathecal injection for 28 days starting 15 min after SCI. On the following day after SCI, CTP was treated to mice once a day for 4 weeks by gavage. **(A)** The BBB scores at 1, 7, 14, 21, and 28 days were shown for all groups of mice. **(B)** Water content in spinal cord of mice at 28 days post-Catalpol treatment. **(C and D)** TUNEL staining of neuronal apoptosis. **(E and F)** The contents of MDA and SOD were measured by commercial kits. **(G–J)** The inflammatory cytokines including TNF-α, IL-1β, IL-6 and IL-10, were evaluated by ELISA assays. Data represent the mean ± SD of three independent experiments. *p < 0.05, **p < 0.01 vs. SCI group; ##*p* < 0.01 vs. SCI + CTP group.

To clarify the role of miR-142 in CTP induced protective effects, the SCI mice treated with antagomiR-142/antagomir-NC via intrathecal injection, followed by CTP treatment. BBB score analysis showed that the improvement of functional recovery caused by CTP treatment was impaired by antagomiR-142 (*p* < 0.0001, Figure 4A). Significantly increased water contents (*p* = 0.0100) and the number of TUNEL positive cells (*p* = 0.0005) in spinal cord tissues were found after administering with antagomiR-142 compared with the CTP-treated SCI mice group (Figures 4B–D). CTP treatment significantly increased the levels of SOD (*p* = 0.0015), decreased the levels of MDA (*p* = 0.0036) compared with SCI group (Figures 4E,F). However, the improvement of CTP in SCI induced oxidative damage was attenuated by antagomiR-142. In addition, the suppressive effect of CTP on inflammatory response (TNF-α, *p* = 0.0004; IL-6, *p* = 0.0040; IL-1β, *p* = 0.0002; IL-10, *p =* 0.0023) was also reversed by antagomiR-142 (Figures 4G–J). According to our results, CTP exhibits the protective effects of CTP on SCI mice through regulating miR-142 expression.

### Catalpol Reduces Apoptosis, Inflammation and Oxidative Stress in Spinal Cord Injury Cell Model

**FIGURE 5:**
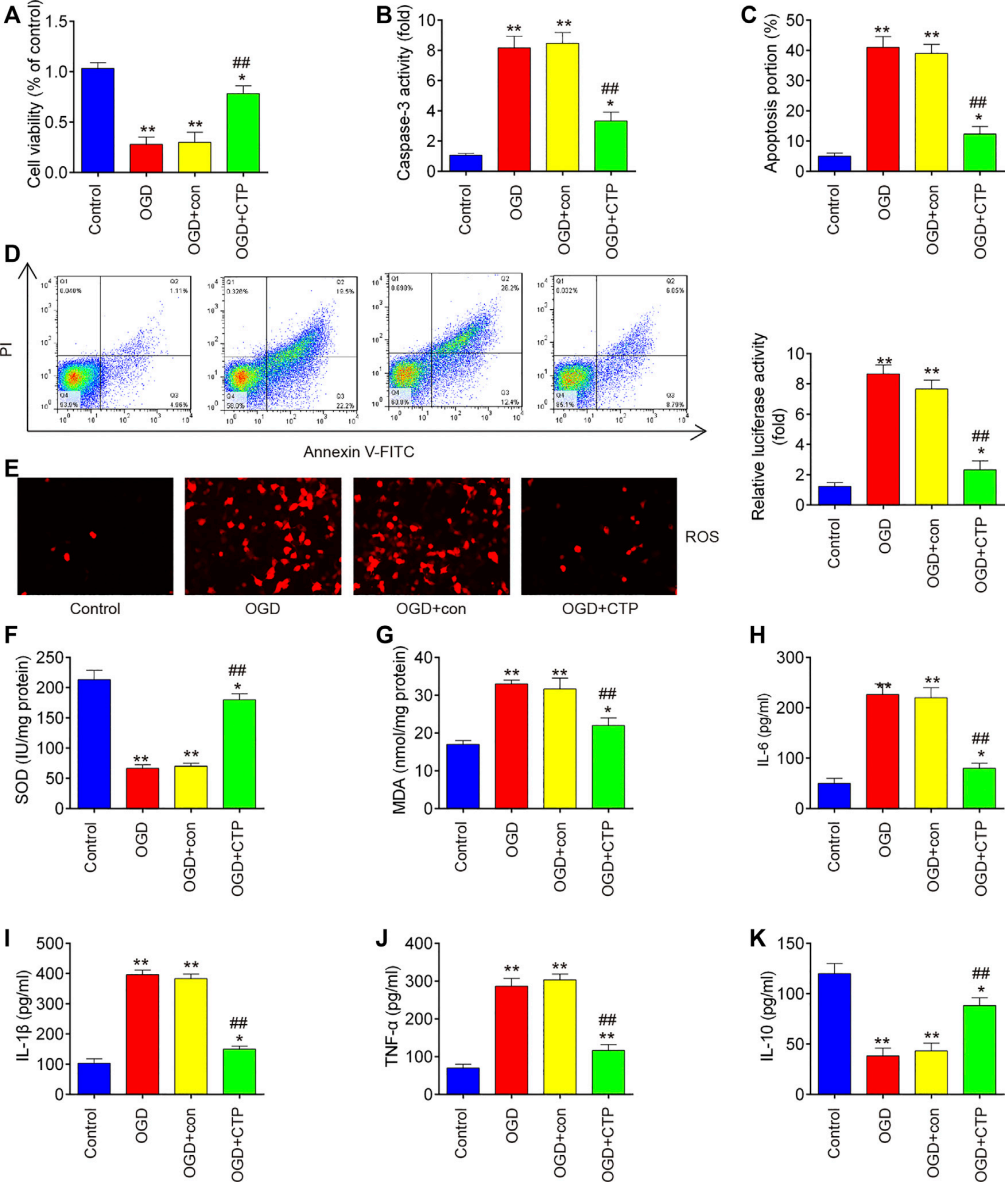
Catalpol reduces apoptosis, oxidative stress and inflammation in SCI cell model. **(A)** Cell viability was assessed using CCK-8 assay. **(B)** Activity of caspase-3 was measured using a caspase-3 Activity Assay kit. **(C and D)** Apoptosis was measured using flow cytometry. **(E)** ROS generation was calculated using 2,7-DCF-DA staining kit **(F and G)** The contents of MDA and SOD were measured by commercial kits. **(H–K)** The inflammatory cytokines including TNF-α, IL-1β, IL-6 and IL-10, were evaluated by ELISA assays. Data represent the mean ± SD of three independent experiments. *p < 0.05, **p < 0.01 vs. Control group; ##*p* < 0.01 vs. OGD/G group.

To explore the molecular basis of CTP in SCI-induced apoptosis, inflammation and oxidative stress, SCI cell models using immortal BV-2 murine microglial cells were established through OGD/R induction as previously described (Wang et al., 2020). Significant reduction of cell viability (*p* = 0.0001), augmented caspase three activity (*p* < 0.0001) and increased apoptotic rate (*p* < 0.0001) were observed in BV-2 cells exposed to OGD/R, which indicates that neuronal injury was successfully induced by OGD/R (Figures 5A–D). Meanwhile, CTP treatment markedly improved cell viability (*p* = 0.0012), reduced the activity of caspase-3 (*p* = 0.0009) and cell apoptosis (*p* = 0.0004) in OGD/R treated-BV-2 cells, suggesting that CTP could alleviate OGD/R induced neuronal injury (Figures 5A–D). Furthermore, the influence of CTP on oxidative stress and inflammatory response were assessed. As shown in Figures 5E–G, the levels of ROS (*p* < 0.0001) and the activity of SOD (*p* < 0.0001) was significantly decreased, while the activity of MDA (*p* < 0.0001) was markedly increased in OGD/R group compared with control group. However, OGD/R induced oxidative damage was alleviated by CTP treatment (ROS, *p* = 0.0002; SOD, *p* < 0.0001; MDA, *p* = 0.0010). In addition, the levels of IL-6 (*p* = 0.0002), IL-1β (*p* < 0.0001) and TNF-α (*p* < 0.0001) were significantly increased, while the level of IL-10 (*p* = 0.0004) was markedly decreased in OGD/R group compared with control group. In contrast, OGD/R induced inflammatory response (TNF-α, *p* = 0.0003; IL-6, *p* = 0.0004; IL-1β, *p* < 0.0001; IL-10, *p =* 0.0013) was attenuated by CTP treatment (Figures 5H–K). All these data suggest that CTP reduces OGD/R induced cell apoptosis, inflammation and oxidative stress *in vitro.*

### Catalpol Improved OGD/R Induced Neuronal Injury by Targeting MiR-142 *in Vitro*

**FIGURE 6:**
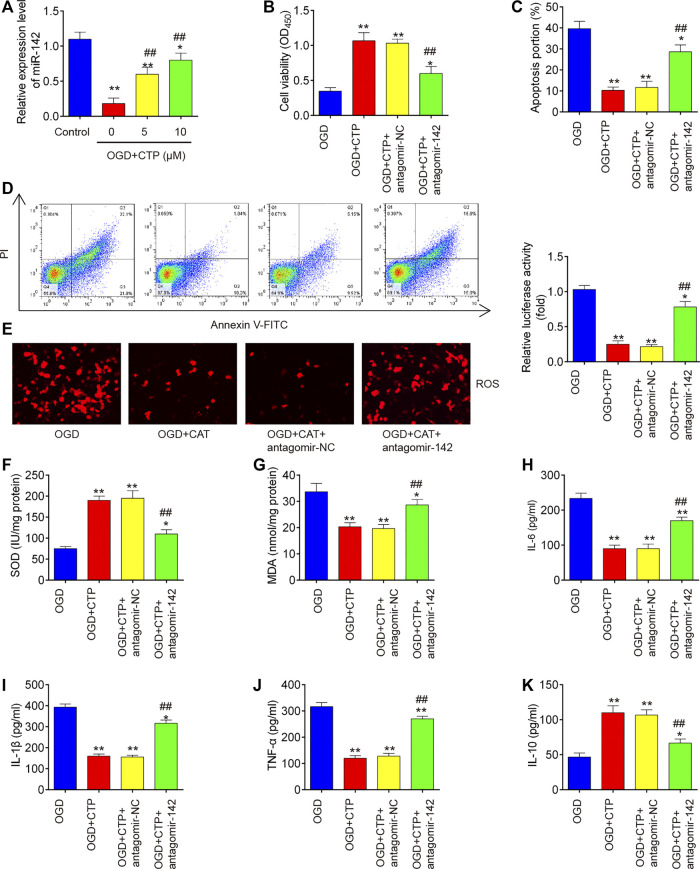
Catalpol improved OGD/R induced neuronal injury by upregulating miR-142 *in vitro.* BV-2 cells were transfected with antagomiR-142 in the presence with CTP (0, 5 and 10 μM), following subjected to OGD/R. **(A)** The expression of miR-142 was determined by qRT-PCR. **(B)** Cell viability was assessed using CCK-8 assay. **(C and D)** Apoptosis was measured using flow cytometry. **(E)** ROS generation was calculated using 2,7-DCF-DA staining kit. **(F and G)** The contents of MDA and SOD were measured by commercial kits. **(H–K)** The inflammatory cytokines including TNF-α, IL-1β, IL-6 and IL-10, were evaluated by ELISA assays. Data represent the mean ± SD of three independent experiments. *p < 0.05, **p < 0.01 vs. OGD/R group; ##*p* < 0.01 vs. OGD/R + CTP group.

To further investigate whether the protective effects of CTP in SCI *vitro* model is mediated by miR-142, BV-2 cells were transfected with antagomiR-142 in the presence with CTP (0, 5 and 10 μM), following subjected to OGD/R. QRT-PCR assay showed that miR-142 was dose-dependently increased after CTP treatment in OGD/R treated BV-2 cells (*p* = 0.0046, Figure 6A). Functionally, knockdown of miR-142 by antagomiR-142 impaired the improvement of CTP on cell viability in OGD/R treated BV-2 cells (*p* = 0.0061, Figure 6B). Moreover, the inhibitory effect of CTP on cell apoptosis was reversed by knockdown of miR-142 in OGD/R treated BV-2 cells (*p* = 0.0009, Figures 6C,D). Furthermore, CTP treatment markedly reduced ROS production (*p* = 0.0005, Figure 6E) and MDA activity (*p* = 0.0050, Figure 6G), and increased SOD activity (*p* = 0.0006, Figure 6F), whereas these effects of CTP were reversed by knockdown of miR-142. Similarly, CTP treatment significantly decreased TNF-α (*p* < 0.0001), IL-6 (*p* = 0.0006), IL-1β (*p* = 0.0001) protein expression levels, and increased the IL-10 (*p* = 0.0029) expression in OGD/R treated BV-2 cells, while these effects of CTP was reversed by knockdown of miR-142 (Figures H–K). Collectively, CTP improved OGD/R induced neuronal injury by targeting miR-142 *in vitro.*

### HMGB1 Is a Direct Target of MiR-142 *in Vivo* and *in Vitro*

**FIGURE 7:**
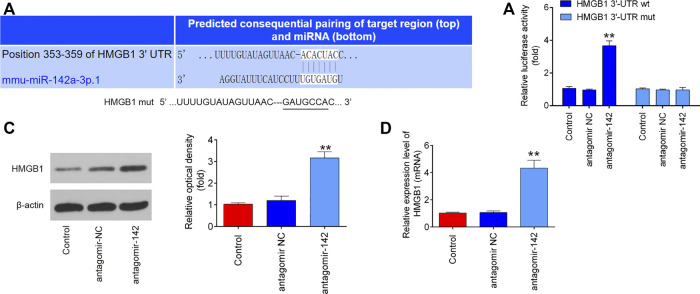
HMGB1 is a direct target of miR-142. **(A)** miR-142-binding sequences in the 3′-UTR of HMGB1 and mutated sites in 3′-UTR of HMGB1. **(B)** The relative luciferase activity of HMGB1-2 wild type (wt) or mutant (mut) 3′-UTR in BV-2 cells following transfection with antagomiR-142 and antagomiR NC as exhibited (n = 3). **(C and D)** The protein and mRNA expressions of HMGB1 were detected by western blot and qRT-PCR after treatment with antagomiR-142 in BV-2 cells. All measurement data which were expressed as mean ± standard deviation and analyzed by one-way analysis of variance. **p < 0.01 vs. antagomiR-NC group.

To further investigate how miR-142 participated in the protection of CTP on SCI, we performed PicTar version 2007 (https://pictar.mdc-berlin.de/) and TargetScan release 7.0 (http://targetscan.org/) to predict the targets of miR-142. According to bioinformatics analysis, we found that HMGB1 3′-UTR had a binding site with miR-142 (Figure 7A). To verify whether HMGB1 is a direct target of miR-142, a dual-luciferase reporter assay was performed. As shown in Figure 7B, miR-142 inhibition significantly increased the luciferase activity of wt HMGB1 3′-UTR in BV-2 cells (*p* = 0.0031), while the luciferase activity of mut HMGB1 3′-UTR had no significant change. Subsequently, western blot and qRT-PCR analysis indicated that miR-142 knockdown markedly increased HMGB1 expression in BV-2 cells at protein (*p* = 0.0002) and mRNA (*p* = 0.0006) levels (Figures 7C,D). All these data indicate that miR-142/HGMB1 axis may play an important role in the protection of CTP on SCI.

### Catalpol Inhibits the HMGB1-Mediated Activation of the TLR4/NF-κB Pathway Through Upregulating MiR-142 in Spinal Cord Injury Cell Model

**FIGURE 8:**
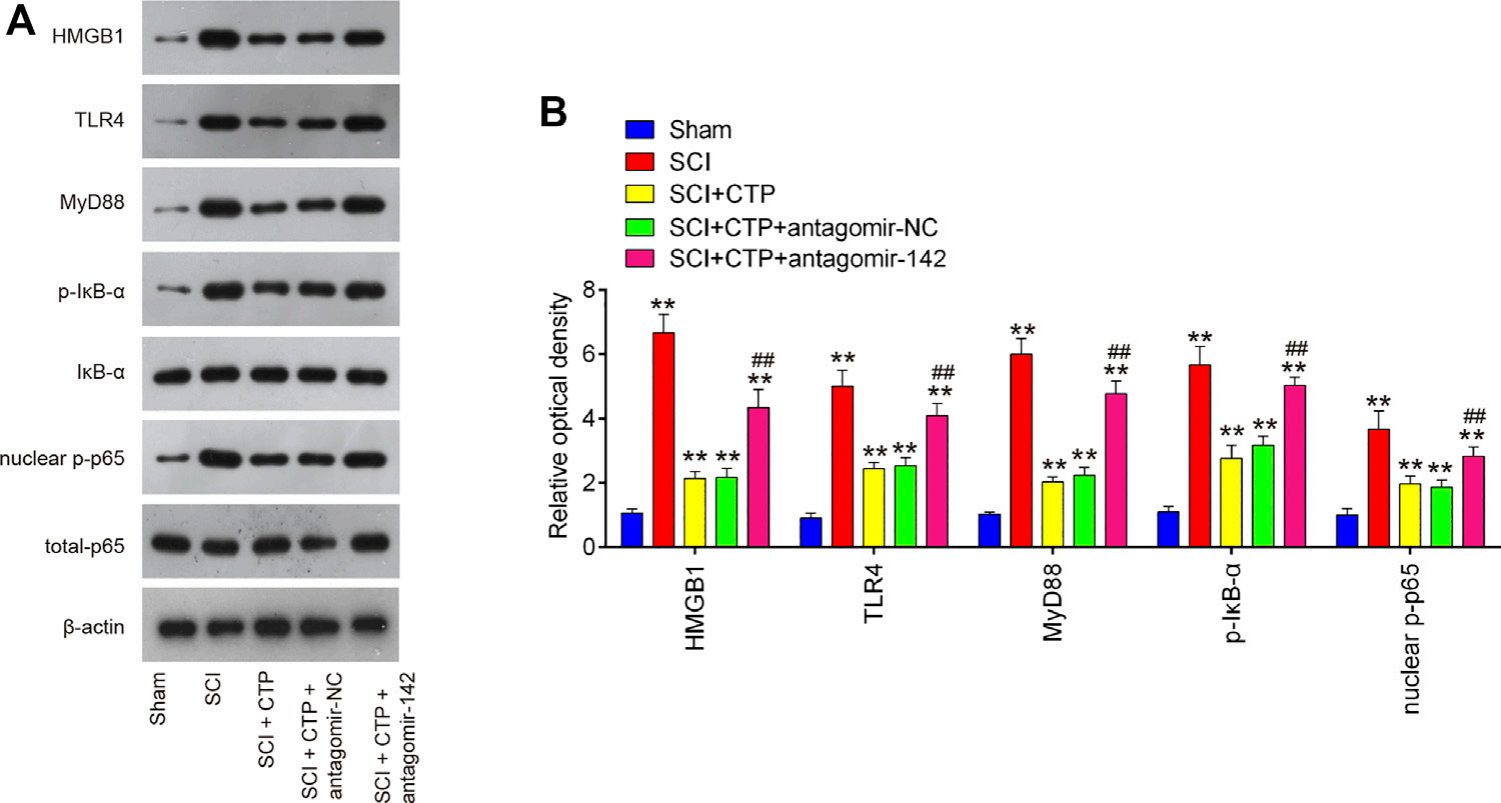
CTP blocked the activation of HMGB1/TLR4/MyD88/NF-κB signaling pathway via regulating miR-142. The mice were subjected to SCI and then treated with antagomiR-142/antagomir-NC via intrathecal injection starting 15 min after SCI. On the following day after SCI, CTP was treated to mice once a day for 4 weeks by gavage. **(A)** The protein expressions of TLR4, HMGB1, MyD88, *p*-IκB-α and nuclear-p-p65 were detected by western blot. **(B)** Bands were semi-quantitatively analyzed using ImageJ software, and normalized to β-actin. Data represent the mean ± SD of three independent experiments. *p < 0.05, **p < 0.01 vs. Sham group; ##*p* < 0.01 vs. SCI + CTP group.

**FIGURE 9:**
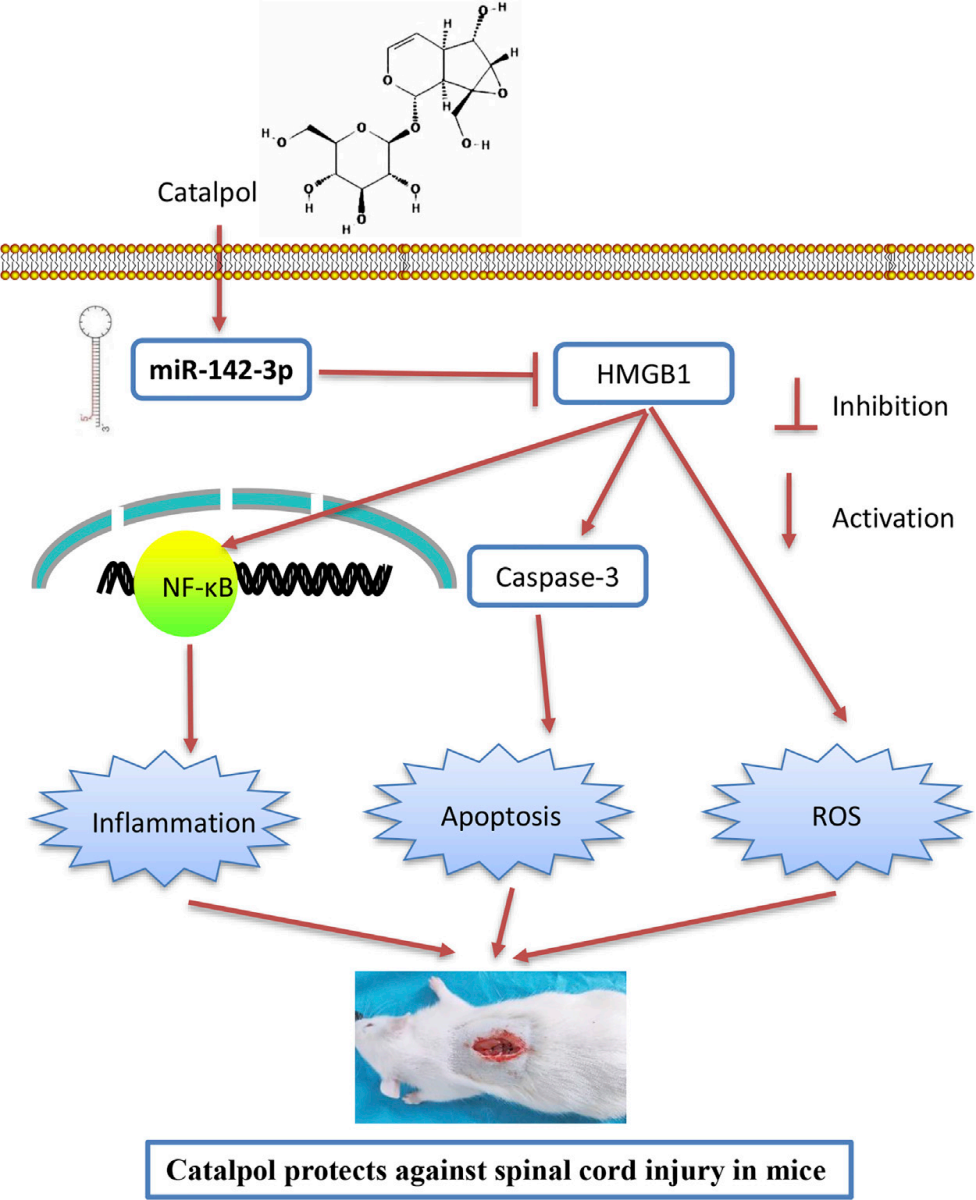
Schematic diagrams showing that CTP regulates HMGB1 expression by miR-142, thereby inhibiting the activation of the TLR4-MyD88-NF-κB signaling pathway, resulting in the suppression of inflammatory response, cell apoptosis and oxidative stress, finally leading to the improvement of SCI.

As we known, HMGB1 through its receptors such as TLR4 activated NF-κB signaling pathway, which contributes to apoptosis, oxidative stress and inflammatory response in SCI (Chen et al., 2011; Yang et al., 2013; Yu and Qian, 2020). Thus, we hypothesized whether CTP improves SCI by targeting miR-142/HMGB1/TLR4/NF-κB pathway. The results showed that the protein expression levels of HMGB1 (*p* < 0.0001), MyD88 (*p* < 0.0001), TLR4 (*p* = 0.0002), *p*-IκB-α (*p* = 0.0002) and p-p65 (*p* = 0.0016) were significantly increased in SCI mice, whereas CTP treatment markedly reduced these proteins (HMGB1, *p* = 0.0003; MyD88, *p* = 0.0002; TLR4, *p* = 0.0012; *p*-IκB-α, *p =* 0.0020; p-p65, *p* = 0.0095) in SCI mice. As expected, these inhibitory effects of CTP on these proteins were reversed by miR-142 knockdown (Figures 8A,B). All these results indicated that catalpol protects against spinal cord injury in mice through regulating microRNA-142-mediated HMGB1/TLR4/NF-κB signaling pathways (Figure 9).

## Discussion

In this study, we observed that CTP improved functional recovery, reduced the neuronal apoptosis, inhibited inflammation and oxidative stress in SCI mice. Additionally, miR-142 was up-regulated by CTP, and inhibition of miR-142 reversed the protective effects of CTP on SCI mice. Interestingly, HMGB1, one important upstream of TLR/NF-κB pathway, was verified as a direct target of miR-142. Besides, miR-142-mediated activation of HMGB1/TLR/NF-κB pathway might be involved in the property of CTP. Hence, our findings indicate that CTP improves SCI through miR-142/HMGB1/TLR/NF-κB pathway, suggesting that CTP could act a potential therapeutic agent in the SCI treatment.

The protective effect of CTP has been widely reported in existing researches, and these protective effects may be attributed to the inhibition of apoptosis, inflammatory or oxidative response. For example, Chen et al. showed that CTP could protect against podocyte injury by ameliorating apoptosis and inflammation in diabetic nephropathy (DN) (Chen et al., 2019). Zhu et al. reported that the protective effects of CTP against renal ischemia/reperfusion injury could occur through inhibiting the inflammatory response (Zhu et al., 2015). Zhang et al. found that pretreatment with CTP could attenuate LPS-induced acute liver injury in mice through the inhibition of inflammatory and oxidative response (Zhang et al., 2018). Several previous studies demonstrated that CTP has neuroprotective effect to perform its function in the injury of different organs. For instance, in spontaneously hypertensive mice (SHR), CTP can effectively ameliorate hyperactive and impulsive behavior, improve spatial learning and memory (Yuan et al., 2019). Wang et al. found that CTP relieved MPTP-triggered oxidative stress, and the occurrence of chronic inflammatory reaction in the mouse model of Parkinson’s disease (PD) (Wang et al., 2019a). However, whether CTP exhibits a beneficial effect on SCI is unclear. In the present study, we investigated the effects of CTP on SCI, and found that CTP could improve the functional recovery, alleviated apoptosis, inflammatory and oxidative response in SCI mice, indicating the CTP has a potential therapeutic effect in SCI.

Growing evidence demonstrated that certain miRNAs are involved in the complex pathological process of SCI, including apoptosis, inflammation and oxidative response (Xie et al., 2018; Lin et al., 2019), while the effects of some traditional Chinese herbal medicines that operate through regulating miRNAs expression have also been reported. For example, Ginsenoside Rb1 (GRb1), a major ingredient of ginseng, has been shown to improve SCI through reducing activated microglia-induced neuronal injury via miR-130b-5p/TLR4/NF-κB axis (Wang et al., 2020). He et al. found that Zhenbao Pill that consists of 29 Chinese herbal medicines had a protective effect on the nerves of SCI mice by regulating miR-214/HSP27 axis (He et al., 2018). With regard to CTP, it exerts inhibitory effects on the progression of several inflammatory diseases through regulating miRNAs, including colitis and stroke (Xiong et al., 2017; Zhu et al., 2019a). In our study, the action mechanism of CTP in SCI was explored by analyzing miRNAs expression profiles. The experiment results revealed that miR-142 expression was enhanced in CTP-treated SCI mice, indicating that miR-142 might be linked with the protective effects of CTP on SCI.

Previous studies have shown that miR-142 possesses protective effects in antagonizing the different organs injury. For example, miR-142 exerted neuroprotective effects against cerebral ischemia/reperfusion (I/R) injury through down-regulation of FBXO3 (Li and Ma, 2020). Yang et al. demonstrated that miR-142a-3p alleviated LPS-induced acute lung Injury (ALI) through down-regulation of NF-κB signaling pathway (Yang et al., 2019). Additionally, miR-142 could improve the sensory function recovery of SCI through elevating cyclic adenosine monophosphate (cAMP) via adenylyl cyclase 9 (AC9), which is abundant in dorsal root ganglia (DRG) (Wang et al., 2015). Moreover, miR-142 was demonstrated to mediate the promoting effects of Sorafenib on sensory conduction function recovery in sciatic nerve conditioning injury (SNCI) in mice (Wang et al., 2019b). Therefore, we hypothesized that miR-142 may be involved in the protective effects of CTP on SCI. In our study, we found that when miR-142 was downregulated by antagomir-142 in CTP treated SCI mice, the functional improvement of locomotor activity after CTP treatment was impaired, as determined by BBB score and spinal cord water content. In addition, the inhibitory effects of CTP on apoptosis, inflammatory and oxidative response were also reversed, suggesting that CTP improved SCI through upregulating miR-142 expression.

HMGB1, a highly conserved nuclear protein, has been reported to be elevated in pre-clinical models of traumatic SCI, where it promotes secondary injury, and inhibition of HMGB1 is an effective strategy improve functional recovery (Papatheodorou et al., 2017). HMGB1 is actively secreted by activated immune cells or passively released from necrotic cells (Scaffidi et al., 2002; Lamkanfi et al., 2010). Once released from cells, HMGB1 can activate innate immune receptors including TLR4, TLR2, subsequently activated NF-κB pathway, which can promote the secretions of inflammatory cytokines, leading to the enlargement of injury area and the damage of anatomic structures (Brambilla et al., 2005; Jimenez-Garza et al., 2005; Fang et al., 2012). After SCI, NF-κB is usually activated in nerve cells and microglia and Kang et al. found that blocked HMGB1/TLR4/NF-κB pathway promoted the functional recovery of SCI mice (Kang et al., 2015). Interestingly, several studies reported that miR-142/HMGB1 axis play a key role in various human cancers, such as glioma and cervical cancer (CC) (Jiang et al., 2017; Li et al., 2018). In our study, HMBG1 was proved to be a target of miR-142, and miR-142 upregulated reduced the expression of HMGB1 at protein and mRNA levels *in vivo* and *in vitro*. Moreover, our data showed that CTP reduced the levels of key TLR4/NF-κB pathway proteins by suppressing miR-142/HMGB1 axis. All data suggest that CTP suppressed HMGB1 via miR-142 to block the TLR4/NF-κB pathway activation and therefore repressed the SCI-induced secondary injury.

## Conclusion

In conclusion, CTP can protect against SCI by ameliorating apoptosis, inflammatory and oxidative response in spinal cord tissues. These protective effects may be attributed to the upregulation of miR-142, which suppressed the expression of HGMB1 to prevent the TLR4/NF-κB activation. Therefore, CTP could be a promising drug for the treatment of SCI patients in future.

## Data Availability

Microarray dataset was obtained from GEO database (https://www.ncbi.nlm.nih.gov/geo/query/acc.cgi?acc=GSE19890) and the GEO accession number is GSE19890.
