# Supplementary figures and images for: Catalpol Protects Against Spinal Cord Injury in Mice Through Regulating MicroRNA-142-Mediated HMGB1/TLR4/NF-κB Signaling Pathway
Source: Front Pharmacol. 2021 Feb 8;11:630222. doi: 10.3389/fphar.2020.630222 (PMC7898164; doi:10.3389/fphar.2020.630222)

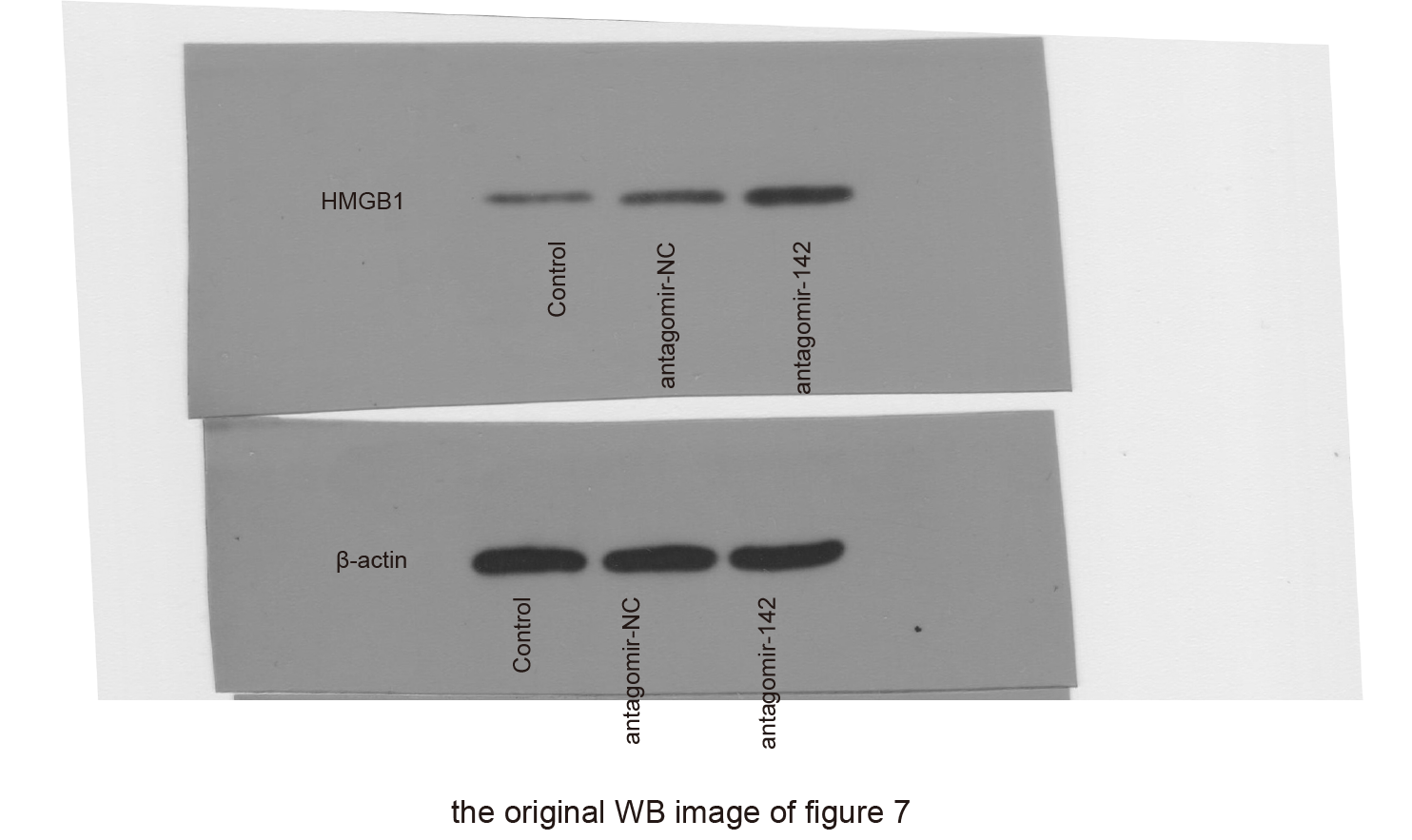

Supplement: Supplementary file 1 [file image1.tif]

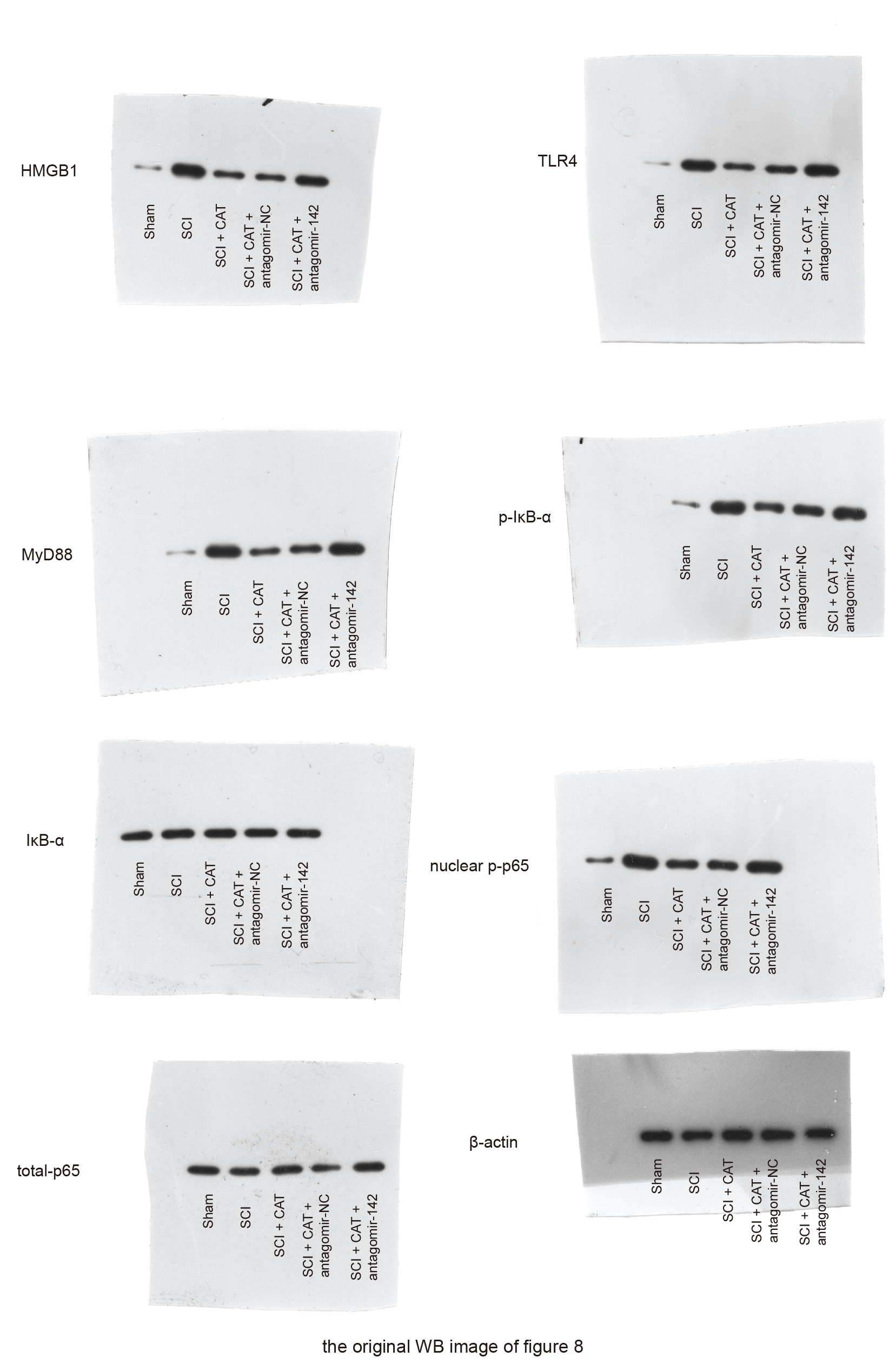

Supplement: Supplementary file 2 [file image2.tif]
